# Patterns of health workforce turnover and retention in Aboriginal Community Controlled Health Services in remote communities of the Northern Territory and Western Australia, 2017–2019

**DOI:** 10.1186/s12960-024-00942-9

**Published:** 2024-08-22

**Authors:** Prabhakar Veginadu, Deborah J. Russell, Yuejen Zhao, Steven Guthridge, Mark Ramjan, Michael P. Jones, Supriya Mathew, Michelle S. Fitts, Lorna Murakami-Gold, Narelle Campbell, Annie Tangey, John Boffa, Bronwyn Rossingh, Rosalie Schultz, John Humphreys, John Wakerman

**Affiliations:** 1 Menzies School of Health Research, Charles Darwin University, PO Box 795, Alice Springs, Northern Territory 0871 Australia; 2 Northern Territory Department of Health, Darwin, Northern Territory Australia; 3 Menzies School of Health Research, Charles Darwin University, Darwin, Northern Territory Australia; 4 Top End Population and Primary Health Care, Northern Territory Government, Darwin, Northern Territory Australia; 5 Psychology Department, Macquarie University, North Ryde, New South Wales Australia; 6 Institute for Culture and Society, Western Sydney University, Parramatta, New South Wales Australia; 7 Poche Centre for Indigenous Health and Well-Being, Flinders University, Alice Springs, Northern Territory Australia; 8 Flinders Rural and Remote Health Northern Territory, College of Medicine and Public Health, Flinders University, Darwin, Northern Territory Australia; 9 Ngaanyatjarra Health Service, Alice Springs, Northern Territory Australia; 10 Central Australian Aboriginal Congress, Alice Springs, Northern Territory Australia; 11 Miwatj Health Aboriginal Corporation, Nhulunbuy, Northern Territory Australia; 12 Monash University School of Rural Health, Bendigo, Victoria Australia

**Keywords:** Health workforce, Primary health care, Aboriginal Community Controlled Health Services, Indigenous health services, Remote health, Aboriginal and Torres Strait Islander peoples, Turnover, Retention

## Abstract

**Background:**

Aboriginal Community Controlled Health Services (ACCHSs) in Australia aim to optimise access to comprehensive and culturally safe primary health care (PHC) for Aboriginal populations. Central to quality service provision is the retention of staff. However, there is lack of published research reporting patterns of staff turnover and retention specific to ACCHSs. This study quantified staff turnover and retention in regional and remote ACCHSs in the Northern Territory (NT) and Western Australia (WA), and examined correlations between turnover and retention metrics, and ACCHSs’ geographical and demographic characteristics.

**Methods:**

The study used 2017–2019 payroll data for health workers in 22 regional and remote PHC clinics managed by 11 ACCHSs. Primary outcome measures included annual turnover and 12-month stability rates, calculated at both clinic and organisation levels.

**Results:**

There was a median of five client-facing (Aboriginal health practitioners, allied health professionals, doctors, nurses/midwives, and ‘other health workers’ combined) and two non-client-facing (administrative and physical) staff per remote clinic, at any timepoint. Mean annual turnover rates for staff were very high, with 151% turnover rates at the clinic level and 81% turnover rates at the organisation level. Mean annual turnover rates for client-facing staff were 164% and 75%, compared to 120% and 98% for non-client-facing staff, at clinic and organisational levels, respectively. Mean 12-month stability rates were low, with clinic-level stability rates of only 49% and organisation-level stability rates of 58%. Mean annual clinic-level turnover rates were 162% for non-Aboriginal staff and 81% for Aboriginal staff. Both workforce metrics were moderately to highly correlated with the relative remoteness of clinics, size of regular clients serviced, and average annual headcount of employees in each clinic (*p* values < 0.01).

**Conclusions:**

Participating ACCHSs in remote NT and WA have very high turnover and low retention of healthcare staff. Overall, clinic-level turnover rates increase as distance from regional centres increases and are lower for Aboriginal staff, suggesting that greater employment of Aboriginal staff could help stabilise staffing. Improved retention could reduce burden on ACCHSs’ resources and may also support quality of service delivery due to improved cultural safety and continuity of care.

**Supplementary Information:**

The online version contains supplementary material available at 10.1186/s12960-024-00942-9.

## Introduction

Timely and continued access to appropriate primary health care (PHC) is critical for the overall well-being of individuals [1]. Access to PHC decreases with increasing geographical remoteness [2], and a key contributor to poor access in the remote context is the limited availability of a stable health workforce [3]. The quality of care and patient outcomes are improved when care is provided by the same PHC professional over time [4]. In rural and remote contexts, continuity of patient care and the development of trusting relationships between patients and their PHC providers may be significantly impeded by the constant movement of health workers into and out of these communities [5, 6]. An unstable workforce has a negative impact on patient engagement with local PHC services [7]. Decreased access to PHC is associated with suboptimal health outcomes, such as increased hospitalisations [8].

In Australia, Aboriginal Community Controlled Health Services (ACCHSs) and state or territory governments operate PHC clinics which provide services to remote Aboriginal communities. ACCHSs aim to improve access to comprehensive PHC for Aboriginal populations in Australia, and as the name suggests, are governed by boards comprising, mainly, local community members, which reflects community ownership of local health services [9]. ACCHSs are underpinned by a model of PHC tailored to meet the needs and expectations of local communities [10], thereby reducing the most common barriers to healthcare access for Aboriginal people relating to cultural appropriateness and acceptability [11] and tackling the adverse effects of disempowerment and racial discrimination on their health [12]. ACCHSs seek to employ staff from local Aboriginal communities. In 2021–22, among the PHC services funded by the Commonwealth Indigenous Australians’ Health Program, 52% of total full-time equivalent positions in ACCHSs were held by Aboriginal people, compared to 38% in non-ACCHSs [13]. Previous research shows that in remote clinics operated by the Northern Territory (NT) Government Department of Health (DoH), the likelihood of staff turnover is lower in Aboriginal staff compared to non-Aboriginal staff; however, there are no comparable data for remote ACCHSs [14].

Qualitative research notes that increasing health workforce stability (and reducing turnover) are essential aspects of strengthening the ACCHS workforce [15], and staff turnover and retention are amongst the most important service delivery issues experienced by ACCHSs [16]. Two key measures of turnover and retention relevant to the Australian rural and remote workforce context are the annual turnover rate and 12-month stability rate [17]. In NT DoH remote clinics the mean annual turnover rates of nurses and Aboriginal health practitioners (AHPs) were 148% and 79%, respectively, while mean 12-month stability rates were 48% and 76%, respectively [14].

The objectives of this study, therefore, were to: a) quantify the most recent pre-COVID-19 pandemic (2017–2019) PHC staff turnover and retention rates in 11 ACCHSs in regional and remote NT and Western Australia (WA); and b) examine the correlations between workforce turnover and retention metrics and ACCHS clinic characteristics, such as geographical remoteness, regular client service population, and clinic workforce size.

## Methods

### Study setting

The study is set in two Australian jurisdictions, NT and WA, which cover over 50% of Australia’s landmass (4 million km^2^). There are a total of 39 ACCHSs providing services in these two jurisdictions [18], of which 11 participated in this study. Participating ACCHSs provided PHC services to 30 communities (via local clinics or outreach services), and collectively serviced around 63,500 Aboriginal people between 2017 and 2019. The ACCHSs serviced regional, remote or very remote communities.

### Study data

The study used payroll system data extracted from the administrative databases of the participating ACCHSs, from 1 January 2017 to 31 December 2019. The data comprised de-identified individual-level information on all employees who received payments directly from the ACCHSs’ payrolls. The participating ACCHSs also recruit some employees through employment agencies, for example, remote area nurses and midwives (hereafter referred to as RANs) and general practitioner (GPs) locums. Those RANs sourced via agencies generally receive payments directly from their recruiting agency rather than through the ACCHSs’ payrolls. While the nature of agency employment model innately contributes to turnover in health services, data about agency-employed RANs were not included in the analyses as they were not consistently available in the payroll data. The measures reported in this study, therefore, relate only to directly employ permanent and casual (non-agency and non-locum) staff.

The structure of the payroll system and corresponding software differed between the ACCHSs. As a result, there were considerable variations in the type and nature of data elements in the payroll data provided for this study. The data cleaning procedure included ensuring key payroll variables were consistently prepared for the analyses (see Supplementary file for details). Staff were assigned to one of seven employment categories based on their role description: (1) administrative (e.g., reception staff, finance officers, human resource staff, policy officers, managers, etc.); (2) AHPs (i.e., healthcare practitioners registered with the Aboriginal and Torres Strait Islander Health Practice Board of Australia); (3) allied health professionals; (4) doctors; (5) RANs; (6) other health workers such as community liaison officers, health promotion officers, counsellors, etc.; and (7) physical grades such as drivers, cleaners, gardeners, tradespeople, maintenance staff, etc. For analyses, AHPs, allied health professionals, doctors, nurses, and other health workers were further grouped as ‘*client-facing*’ staff; administrative and physical categories were grouped as ‘*non-client-facing*’.

### Analysis

Turnover and retention metrics were calculated at two levels: (a) community or *clinic level* and (b) service-wide or *organisation level*. Organisation-level analysis included 11 participating ACCHSs, while analysis at the clinic level was restricted to 22 clinics serviced by the ACCHSs. The decision to exclude eight clinics for the clinic-level analysis was to ensure that only clinics that were actively running and staffed for the entire study period, were included. For example, there were some clinics in the participating ACCHSs that had transitioned from NT DoH to community-controlled administration during the study period, and therefore, only had partial data available for analysis (see Supplementary file for details). Excluding such clinics enabled reasonable comparison among the ACCHSs when grouped into categories for further analyses.

Employee exits were defined at the clinic and organisation levels, following the approach by Russell et al*.* [14]. Exits were measured at the clinic level (clinic-level turnover) if an employee left one of the 22 clinics in which PHC services were being delivered for a period of more than 12 weeks. Employee movement between different clinics, even while remaining employed by a given ACCHS, was thus considered an exit, according to this definition. Second, an exit was measured at the organisation level if an employee ceased (for more than 12 weeks) employment with the ACCHS. In this instance, movements between clinics while remaining employed by a single ACCHS were not measured as an exit. Defining exits at these two levels facilitates better understanding of the direct impact of staff turnover on interpersonal continuity of care for patients attending individual clinics (clinic level) and overall disruption to the functioning of the ACCHS (organisation level).

Health workforce supply was measured using two metrics at each of clinic and ACCHS levels—total number of unique employees and average headcount, defined as follows:

1. Total number of unique employees = number of individuals employed during 3 years
2. Average headcount = average number of individuals employed at any given point in time during 3 years

Two key workforce turnover and stability metrics were calculated:

1 $$\text{Mean annual turnover rate }\left(\text{\%}\right) = \frac{(\text{total number of exits in the }36\text{ month period}/3)}{\text{average headcount during the }36\text{ month period}} \times 100$$

2 $$\text{Mean }12-\text{month stability rate }\left(\text{\%}\right) = \frac{\sum \left\{\frac{\text{number of employees at start of each year who remain employed }12\text{ months later}}{\text{number of employees at start of each year}} \times 100\right\}}{3}$$

Summary statistics for each key workforce metric were analysed at the organisation and clinic level for all staff, and according to their employment category (i.e., client-facing and non-client-facing staff) and Aboriginal status (i.e., Aboriginal and non-Aboriginal staff). Analyses were conducted by Aboriginal regular client service populations (hereafter referred to as ‘service population’) and by distances between the remote clinic and the nearest regional centre. The service population were defined as those Aboriginal clients who attended an ACCHS PHC clinic three or more times in the 24 months immediately preceding each study year (2017–2019) [19] and were estimated using ACCHSs’ Communicare electronic medical records of PHC utilisation during the period 2015–2018. Service population size of ACCHSs was classified into three categories (≤ 2000, 2001–10000, > 10,000) and for clinics, into six categories (≤ 500, 501–1000, 1001–1500, 1501–2000, 2001–10000, > 10,000). ‘Regional centres’ were defined as those with acute care hospital facilities. Distances to the nearest regional centre were measured in kilometres using straight-line distances in Google Maps. At the organisation level, ACCHSs were described as ‘regional’ or ‘remote’ based on the proximity to the nearest regional centre. An ACCHS was categorised as ‘remote’ if > 50% of the communities it serviced were further than 50 km from the nearest regional centre. For the clinic-level summary, distance to the nearest regional centre was distributed into four categories (≤ 50, 51–200, 201–500, > 500).

Summary statistics were reported as means with standard deviations (SD) or medians with interquartile ranges (IQR). Mann–Whitney *U* test was used to compare key workforce metrics between the two staff groups analysed—client-facing vs. non-client-facing and Aboriginal vs. non-Aboriginal. Spearman rank correlation coefficients (ρ) were used with a statistical significance test to explore associations between clinic-level key workforce metrics and community characteristics. Absolute value of ρ (|ρ|) indicated the strength of the correlation. A *p* value of < 0.05 was considered statistically significant and has not been adjusted for multiple inferences as this is primarily a descriptive study.

## Results

The median service population of the 22 clinics over the 3-year study period was 1110 (IQR 620, 1758). The median straight-line distances from the clinics to the nearest regional centre was 185 km (IQR 9, 467).

### Clinic-level turnover and retention

At any point in time in a given year, the median number of client and non-client-facing staff working in a remote clinic (i.e., clinics > 50 km from the nearest regional centre, as previously defined) was 5 (IQR 4, 8) and 2 (IQR 1, 6), respectively.

Overall, 1690 staff ceased providing services in the 22 clinics during the study period. The mean annual turnover rate was estimated as 151% (± 124.2) for all staff (Table 1); for client-facing staff, this was 164%, compared to 120% for non-client-facing staff (Table 1). The 12-month stability rates averaged 49% (± 17.9) overall, with similar means for client-facing (50%) and non-client-facing (49%) staff (Table 1). There was no statistically significant difference in annual turnover and 12-month stability rates between the two staff groups.

**Table 1:** Summary of clinic-level staff metrics, by regular client service population size

Regular client service population size category	No. of clinics	% Mean annual turnover (SD)			% Mean 12-month stability (SD)		
		All staff	Client facing staff	Non-client facing staff	All staff	Client facing staff	Non-client facing staff
≤ 500	4	376.3 (81.4)	420.3 (133.8)	230.4 (126.1)	25.9(11.1)	23.8 (11.5)	25 (9.6)
501–1000	7	126.3 (73.7)	131.7 (79.1)	106.4 (62.9)	52.2 (1.4)	51.9 (1.3)	44.2 (1.8)
1001–1500	4	94.9 (40.5)	98.5 (11.5)	114.2 (106.3)	56 (15)	53.5 (14.1)	50.2 (23)
1501–2000	3	107.1 (51.1)	121.2 (82.1)	102.8 (5.9)	51.1 (9)	51.5 (15.9)	44 (11.8)
2001–10000	2	66.1 (6)	72.9 (7.2)	58.6 (4.6)	57.7 (0.8)	51.9 (1.4)	63.8 (0.2)
> 10,000	2	48.7 (3.7)	52.5 (2.9)	41.2 (7.3)	67.9 (2.3)	69.1 (5.9)	72.3 (5.8)
Total	22	150.9 (124.2)	164.2 (144.6)	119.6 (93.4)	49.4 (17.9)	49.9 (16.9)	49.3 (21)

Total mean calculated at an aggregate level for all clinics

*SD* standard deviation

Clinics in the category representing those that are furthest from the nearest regional centre had the highest mean annual turnover rates among all staff at 355% (± 85) and the lowest 12-month stability rates at 26% (± 11.1), compared to clinics in other categories (Table 2). The trends in the metrics were similar for categories based on number of service clients, where annual turnover rates and 12-month stability rates for clinics representing the smallest category averaged at 376% (± 81.4) and 27% (± 9.9), respectively (Table 1).

**Table 2:** Summary of clinic-level workforce metrics, by Euclidean distance to nearest regional centre

Euclidean distance (in km) to nearest regional centre category	No. of clinics	% Mean annual turnover (SD)			% Mean 12-month stability (SD)		
		All staff	Client facing staff	Non-client facing staff	All staff	Client facing staff	Non-client facing staff
Regional centres and ≤ 50	7	63.9 (18)	77 (32)	50.3 (11.2)	65.2 (8.5)	63 (11.3)	69.2 (7.8)
51–200	4	89.5 (51.3)	78.3 (34.9)	111.3 (79.3)	54.6 (12.7)	58.3 (9.5)	46.2 (21.7)
201–500	6	123.1 (27.9)	130.6 (37.4)	126.3 (79.3)	47.9 (8.3)	47.5 (12.6)	49.9 (13.7)
> 500	5	355.1 (85)	395.2 (128.8)	215.3 (114.3)	27.1 (9.9)	25.7 (10.8)	23.3 (9.1)

*km* kilometres*, SD* standard deviation

**Fig. 1:**
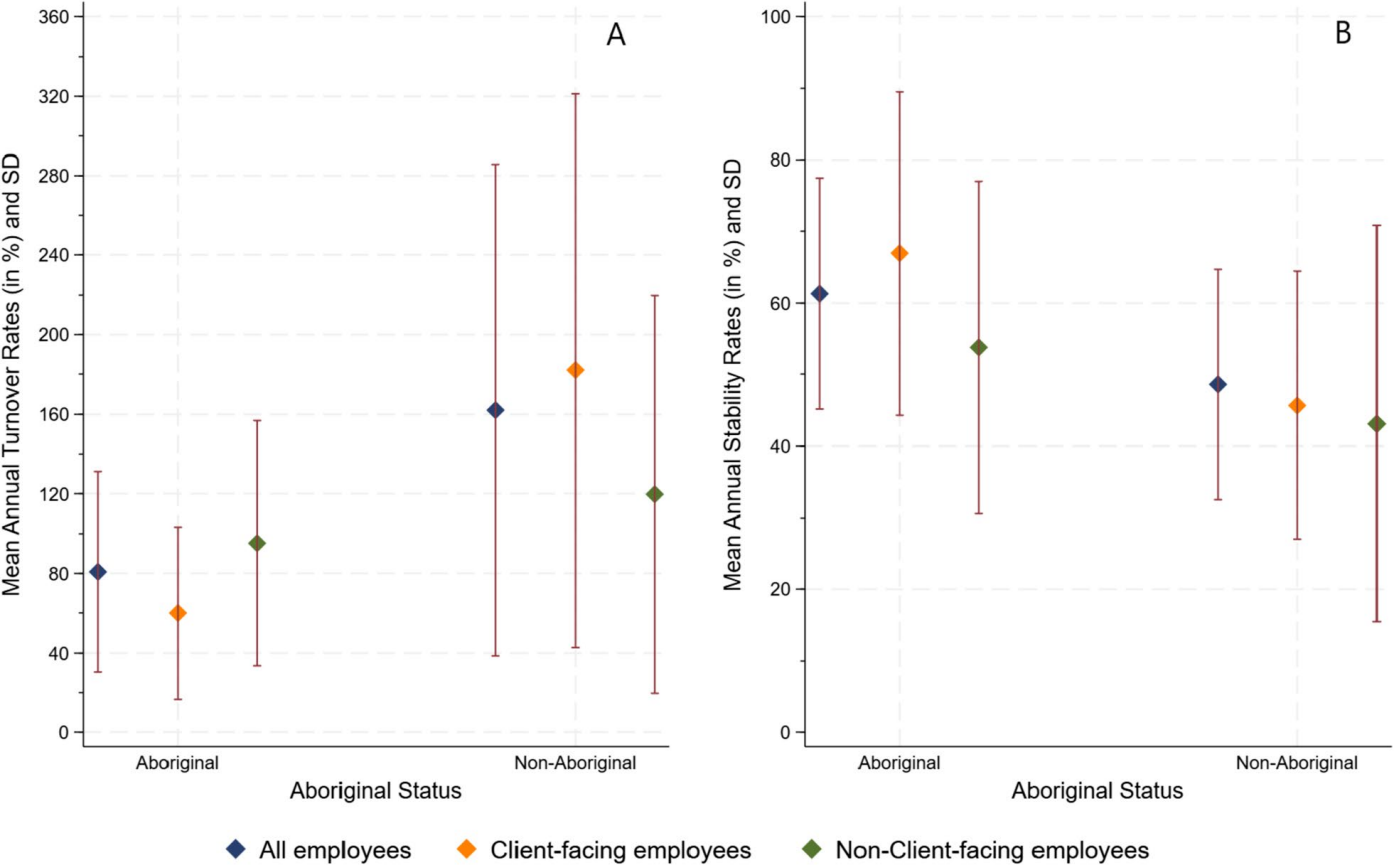
Summary of clinic-level (**A**) turnover and (**B**) stability metrics, by staff employment category and Aboriginal status

The median average headcount of Aboriginal and non-Aboriginal staff working in a clinic was 6 (IQR 4, 18) and 5 (IQR 4, 10), respectively. Noting that five of the 22 clinics did not have any Aboriginal staff employed during the study period, the mean annual turnover rates among Aboriginal staff in the remaining 17 clinics was 81% which was significantly lower than that of non-Aboriginal staff at 162% (*p* = 0.025) (Fig. 1A). The 12-month stability rates were also significantly different between the two groups (*p* = 0.019), with higher stability among Aboriginal staff at 61%, compared to 49% among non-Aboriginal staff (Fig. 1B and Supplementary file Table S1).

### Organisation-level turnover and retention

There were 1630 exits for all staff from the 11 ACCHSs over the study period. This was an overall annual turnover rate of 81% (± 42.9), while the mean 12-month stability rate for 11 ACCHSs was estimated as 58% (± 13.3) (Table 3). Remote ACCHSs had higher mean annual turnover rates (97%) and lower 12-month stability rates (53%), than regional ACCHSs (Table 4).

**Table 3:** Summary of organisation-level staff workforce metrics, by regular client service population size

Regular client service population size category	No. of ACCHSs	% Mean annual turnover (SD)			% Mean 12-month stability (SD)		
		All staff	Client facing staff	Non-client facing staff	All staff	Client facing staff	Non-client facing staff
≤ 2000	5	99 (57.1)	79.1 (40.6)	148.3 (102.3)	53.9 (18.2)	58.5 (17.9)	45.3 (19.8)
2001–10000	3	80.8 (22.9)	87 (30.5)	69.4 (8.2)	56.2 (18.2)	54.3 (9.9)	59.7 (3.9)
> 10,000	3	51.6 (5.7)	57.4 (8.7)	44.1 (7.2)	66.5 (2.8)	63.1 (4.6)	71.1 (4.6)
Total	11	81.1 (42.9)	75.3 (31.7)	98.4 (81.2)	57.9 (13.3)	58.6 (12.8)	56.3 (17.2)

Total mean calculated at an aggregate level for all ACCHSs

*ACCHS* Aboriginal Community Controlled Health Service, *SD* standard deviation

**Table 4:** Summary of organisation-level staff workforce metrics, by remoteness category

Remoteness category	No. of ACCHSs	% Mean annual turnover (SD)			% Mean 12-month stability (SD)		
		All staff	Client facing staff	Non-client facing staff	All staff	Client facing staff	Non-client facing staff
Regional	4	52.9 (12)	54.8 (17.4)	50.2 (13.5)	67.1 (7.2)	65.5 (10.2)	68.7 (5.5)
Remote	7	97.2 (46.4)	87.1 (32.9)	124.7 (93.1)	52.7 (13.4)	54.6 (13.1)	49.1(17.7)

*ACCHS* aboriginal community controlled health service, *SD* standard deviation

On average, the median number of Aboriginal and non-Aboriginal staff working in a remote ACCHS per year was 6 (IQR 3, 24) and 5 (IQR 4, 78), respectively. The mean annual turnover rates were 88% and 75% for Aboriginal and non-Aboriginal staff, respectively, with comparable 12-month stability rates (Supplementary file Table S2). Neither turnover nor stability rates significantly differed between Aboriginal and non-Aboriginal staff groups at the organisation level.

### Correlation of turnover and retention metrics with clinic characteristics

Correlations between the clinic-level key workforce metrics for staff by employment category and distance to the nearest regional centre, service population size, and average annual headcount were moderate to high (|ρ|= 0.5 − 0.9, *p* values < 0.01). For Aboriginal staff, these correlations were lower in magnitude than for non-Aboriginal staff (|ρ|= 0.2 − 0.7) and statistically significant only for distance to the nearest regional centre. (Table 5). Annual turnover rates were positively correlated with remoteness, while 12-month stability rates were negatively correlated. The direction of association was opposite for service population size and average annual headcount, that is, annual turnover rates were negatively correlated with these indicators and 12-month stability rates were positively correlated. Correlations between the workforce metrics and total unique employees working at each clinic were mostly low or negligible and non-significant.

**Table 5:** Correlations between clinic-level workforce metrics (2017–2019) and health service characteristics

Clinic characteristic	Annual turnover rates					12-month stability rates				
	All staff	Client facing staff	Non-client facing staff	Aboriginal staff	Non-Aboriginal staff	All staff	Client facing staff	Non-client facing staff	Aboriginal staff	Non-Aboriginal staff
Distance to the nearest regional centre	0.87***	0.79***	0.78***	0.50*	0.89***	− 0.85***	− 0.75***	− 0.78***	− 0.72***	− 0.81***
Regular client service population	− 0.76***	− 0.73***	− 0.56**	− 0.24	− 0.68***	0.65**	0.60**	0.61**	0.26	0.66**
Total unique employees actually working during the 3-year period	− 0.25	− 0.18	− 0.40	− 0.05	− 0.17	0.18	0.06	0.32	0.15	0.15
Average annual clinic headcount during the 3-year period	− 0.71***	− 0.62**	− 0.66**	− 0.28	− 0.61**	0.66**	0.57**	0.69**	0.41	0.59**

*p < 0.05, **p < 0.01, ***p < 0.001

## Discussion

Importantly, this study describes the pre-pandemic patterns of turnover and retention of PHC staff in ACCHSs in remote and regional areas of NT and WA. The findings indicate very high clinic-level turnover (mean 151%) and low retention (mean 49%) rates, with similar retention rates among client-facing and non-client-facing staff. While not directly comparable, these rates are consistent with those reported in a previous study of remote health care staff in NT DoH clinics [14]. The rates were considerably higher than what were considered acceptable turnover rates among health workforce, elsewhere, where rates over 20% are considered high [20]. PHC workforce turnover has been shown to be negatively associated with PHC service utilisation, especially in the first 12 months after a usual provider of care leaves, and have positive associations with utilisation of urgent and emergency care [21]. An unstable clinical workforce also compromises the continuity and quality of care generally [22, 23].

There is a substantial difference in the mean turnover and stability rates at clinic and organisation levels, with higher turnover and lower retention seen at the clinic level. This is not unexpected, because ACCHS staff may be moved between clinics within the organisation. Workforce and service needs of remote clinics fluctuate for multiple reasons, and ACCHSs may redeploy staff from a different clinic to temporarily fill a vacancy or to supplement the existing PHC service capacity in another. Staffing patterns comprising considerable workforce movements between clinics may impede the development of rapport between residents and health providers. The impact on interpersonal continuity of care, however, may be mitigated if returning short-term staff and longer-term staff who are relocated within the organisation work in the same clinic, and thus have local knowledge and established relationships with residents [7].

One key finding, which differs from previously reported findings, relates to the correlation between clinic-level workforce metrics and remoteness (as measured by distance to nearest acute care hospital facility) and service population size. In this study annual turnover rates significantly increased with increasing remoteness, ranging from 64% (regional centres) to 355% (the most remote clinics), whereas a recent study amongst NT DoH remote clinics found no significant correlation [14]. This difference in association could be because of greater variation in remoteness of ACCHS clinics included in this study—regionally based to very remote clinics—whereas the NT DoH clinics had less variation in remoteness [14]. Similar gradients associated with the size of service populations, were found. The turnover rate, for example, ranged from 376% for the smallest communities to 49% for the largest services, a greater than sevenfold difference. Given the high costs of staff turnover, particularly in the remote context [16]—e.g., associated with frequent staff recruitment, on-boarding and training, and agency and locums salary to temporarily fill the vacancy—and the current shortfall of funding for these remote clinics, estimated at $80 million per year for the NT [24], the study findings have profound implications for funding remote PHC. It suggests that further research is needed to ensure that an equitable, needs-based funding formula is developed which takes into account not only the burden of disease in these communities but also the higher costs of delivering frontline services, compared to urban or peri-urban populations. Such high turnover rates in the most remote locations also have implications for the re-design of appropriate service delivery models to optimise interpersonal continuity of care, including offering flexible employment arrangements to enhance staff retention.

Another notable finding pertains to clinic-level staff turnover stratified by Aboriginal status. While the analysis did not factor in the proportion of local and non-local Aboriginal staff, relatively lower clinic-level turnover rates among Aboriginal staff (81%), compared to non-Aboriginal counterparts (162%), might reflect favourably on the employment of local Aboriginal community members who are likely to have a greater connection and sense of responsibility and commitment to their community. Employment of local Aboriginal people is critical for ensuring culturally sensitive and appropriate service delivery in remote Aboriginal communities, as they are more familiar with the local cultures and contexts. Aboriginal staff in client-facing roles, in particular, have been shown to have a positive impact on service access, care acceptance and overall experience among Aboriginal patients [11, 25]. Nevertheless, annual turnover rates averaging 81% among remote Aboriginal staff are still considerably higher than average turnover rates in other healthcare settings [20]. However, these high turnover rates highlight the need for future research to better understand and address context-specific factors impacting Aboriginal staff retention, particularly that of local Aboriginal staff. This may include providing increased personal and professional support for Aboriginal health professionals, providing pathways to career advancement, resources to enhance skills and capability, ensuring culturally safe and respectful workplaces, remuneration appropriately reflecting expertise, and equitable provision of non-financial incentives such as subsidised housing [26].

It is important to note that the turnover and retention rates presented in this study are likely to underestimate the actual turnover rates and overestimate stability rates. This was confirmed in a sensitivity analysis with and without agency-employed RANs for one of the participating ACCHS, which included agency RANs paid via their payroll system (specific findings not reported to maintain confidentiality). In that ACCHS agency RANs provided a substantial proportion of PHC services. NT DoH had previously estimated 42% of its RAN workforce were agency-employed [14]. These findings reflect the extent of the reliance on locum and agency staff for PHC services in remote clinics. High use of agency RANs is cost-ineffective, mainly owing to the higher costs associated with their recruitment to fill existing vacancies, such as agency fees, travel, housing, and orientation and induction, which further compounds the already high costs of providing PHC in remote health services [27].

Participating ACCHSs have initiated a range of retention strategies, such as flexible employment conditions, cash retention bonuses and non-financial incentives (such as subsidised housing). Notwithstanding these initiatives, stabilisation of the remote health workforce has remained elusive. Workforce stabilisation could lessen the financial burden on ACCHSs, improve the cultural and clinical competence of staff [28], and reduce the workload by decreasing the need to continually orient and support new colleagues [29]. From patients’ perspectives, stabilisation of the workforce could result in improved interpersonal continuity of care and service quality [23], thereby fewer preventable hospital admissions [30].

The study findings must be interpreted with some caution because of limitations arising from data availability and quality. First, comprehensive individual-level data on ACCHSs’ use of GP locums and agency-employed RANs were unavailable and could not be integrated with the payroll data. This precludes obtaining a complete picture of the actual extent of staff movements on the ground. Limited data and anecdotal information from participating ACCHSs indicate high use of GP locums and agency-employed RANs in remote communities. As such, the presented turnover and retention metrics may have been underestimated and overestimated, respectively. Second, the analyses did not account for variations among staff based on the type of employment contracts, i.e., casuals, fixed-term, or continuing. This means that casual staff who continue to be on the ACCHSs’ payroll but did not have any work hours to log for more than 12 weeks and departures of staff on temporary contracts during the study period would have been counted as an exit, thus contributing to the turnover measure. Thirdly, the small size of some participating ACCHS clinics, where some clinics had as few as one staff on average at any given point, meant that there was statistical instability in these turnover rates because of the small denominator. Finally, the collection, recording and configuration of payroll data varied among the ACCHSs, which required extensive data manipulation to enable comparability, for example, grouping staff into the seven pre-defined employment categories and resolving inconsistent staff employment information. There might have been some errors during data interpretation and manipulation that potentially impacted the findings. However, the research was conducted closely with relevant ACCHS staff to ensure maximum accuracy of information and minimal errors in the process and to validate the findings at each stage of progress.

## Conclusion

This landmark study provides a comprehensive description of the pattern of turnover and retention of healthcare staff in regional and remote ACCHSs in the NT and WA. Turnover is very high in regional centres and extraordinarily high in remote clinics. Overall, clinic-level turnover rates are also higher for non-Aboriginal staff than for Aboriginal staff and increases as distances to regional centres increase. These staffing patterns not only are likely to impose an additional financial and workload burden on remote ACCHSs in terms of increased resources required in relation to frequent staff recruitment and orientation but may also compromise the quality of care and health outcomes for remote Aboriginal community members. These findings have important implications for workforce policy and ensuring the equitable funding of ACCHSs.

### Supplementary Information

Supplementary Material 1

## Data Availability

The data sets generated and analysed during the current study are not publicly available due to identifiability of ACCHSs staff and the need to protect their privacy.

## Abbreviations

ACCHS: Aboriginal Community Controlled Health Services
AHP: Aboriginal health practitioners
DoH: Department of Health
GP: General practitioner
IQR: Interquartile range
NT: Northern Territory
PHC: Primary health care
PHN: Primary Health Network
RAN: Remote area nurses and midwives
SD: Standard deviation
WA: Western Australia
