# Supplementary material for: Patterns of health workforce turnover and retention in Aboriginal Community Controlled Health Services in remote communities of the Northern Territory and Western Australia, 2017–2019
Source: Hum Resour Health. 2024 Aug 22;22:58. doi: 10.1186/s12960-024-00942-9 (PMC11340071; doi:10.1186/s12960-024-00942-9)
Supplement: Supplementary file 1 — Supplementary Material 1 [file 12960_2024_942_MOESM1_ESM.docx]

**Supplementary Methods**

**Data preparation:**

Due to the differences in the structure and elements of the payroll data provided between participating Aboriginal Community Controlled Health Services (ACCHS), the data needed considerable preparation before the analyses. For example, some ACCHSs used different typologies and descriptions for various roles. Also, some staff changed positions over time and moved across employment categories, such as between administrative and clinical roles, such as nurse or doctor. For those staff who switched between employment categories during the study period, we applied the following rules:

1. individuals only employed in roles in any of the five client facing employment categories were always considered client facing staff
2. individuals only employed in any of the two non-client facing employment categories were considered non-client facing staff
3. individuals employed across client facing and non-client facing employment categories (e.g., employed in nurse and administrative categories) were considered client facing staff.

**Analysis:**

The 30 clinics serviced by the 11 participating ACCHSs varied in terms of staff type and size, periods of operation, and governance during the study period. As such, some clinics were excluded when analysing the turnover and retention metrics at the clinic and organisation levels for all staff, and according to their employment category (i.e., client facing and non-client facing staff) and Aboriginal status (i.e., Aboriginal and non-Aboriginal staff). The decision on the exclusions was made to ensure that the health services grouped within each category are analogous with respect to the staff composition which in turn enables reasonable comparison between them.

A total of eight clinics were excluded from the clinic-level analysis. These clinics fell into one of the following three categories:

1. transitioned from NT Department of Health governance to ACCHS governance during the study period
2. did not have any staff in either client facing or non-client facing categories at any point during the study period
3. did not have a clinic providing healthcare services, i.e., those with only a corporate services centre.

For the organisation-level analysis, those services that fell into category three above (i.e., those with only a corporate services centre) were retained, while the remaining were excluded. In total, six clinics were excluded at the organisation-level.

**Supplementary Results**

**Table S1. Summary of clinic-level workforce metrics, by Aboriginal status**

| Workforce metric | Aboriginal staff (n=17 clinics) | | | Non-Aboriginal staff (n=22 clinics) | | |
| --- | --- | --- | --- | --- | --- | --- |
|  | All Aboriginal | Client facing | Non-client facing | All non-Aboriginal | Client facing | Non-client facing |
| Total mean annual turnover (%) (SD) | 80.8  (50.4) | 60  (43.4) | 95.3  (61.7) | 162.2  (123.6) | 182  (139.2) | 119.7  (100.1) |
| Total mean 12-month stability (%) (SD) | 61.3  (16.1) | 66.9  (22.6) | 53.8  (23.2) | 48.6  (16.1) | 45.7  (18.7) | 43.1  (27.7) |

*SD*, standard deviation

**Table S2. Summary of organisation-level workforce metrics, by Aboriginal status**

| Workforce metric | Aboriginal staff (n=10 ACCHSs) | | | Non-Aboriginal staff (n=11 ACCHSs) | | |
| --- | --- | --- | --- | --- | --- | --- |
|  | All Aboriginal | Client facing | Non-client facing | All non-Aboriginal | Client facing | Non-client facing |
| Total mean annual turnover (%)  (SD) | 88  (64.8) | 68.5  (52.1) | 104.2  (79.8) | 74.5  (32.4) | 79.1  (31.3) | 97.2  (127.8) |
| Total mean 12-month stability (%) (SD) | 62.2  (18.9) | 64  (27.2) | 61  (20.9) | 58.5  (9.6) | 58.8  (11.8) | 50.6  (29.5) |

*ACCHS,* Aboriginal Community Controlled Health Service; *SD*, standard deviation
